# CAR T-Cell Therapy in Systemic Lupus Erythematosus: Mechanistic Rationale, Early Clinical Experience, and Future Challenges

**DOI:** 10.31138/mjr.171125.mer

**Published:** 2026-06-11

**Authors:** Sabarinath Mahadevan, Saranya Chinnadurai, Balakrishnan Navaneethakrishnan

**Affiliations:** 1 Institute of Rheumatology, MMC & RGGGH, Chennai, Tamilnadu, India;; 2 Department of Rheumatology, Saveetha Medical College and Hospital, Chennai, Tamilnadu, India;; 3 Consultant Rheumatologist, St Thomas Hospital, Chethipuzha, Chenganaserry, Kerala, India

**Keywords:** chimeric, lupus, stem cell, cytokine release syndrome, immunosuppression, autoimmune diseases

## Abstract

Systemic lupus erythematosus (SLE) remains a challenging multisystem autoimmune disease characterised by relapses and remissions, often necessitating lifelong immunosuppression. Over the past decades, the therapeutic landscape has evolved from broad-spectrum agents such as cyclophosphamide to more targeted biologics including rituximab, belimumab, and anifrolumab—each contributing to incremental improvements in disease control. More recently, cell-based therapies have emerged as a novel strategy to reprogramme immune responses, including haematopoietic stem cell transplantation, bispecific T-cell engager (BiTE) therapy, and chimeric antigen receptor (CAR) T-cell therapy. CAR T-cell therapy represents a potentially transformative approach in SLE, offering the prospect of sustained clinical and immunological remission without ongoing immunosuppression. In this narrative review, we searched the Medline/PubMed, Scopus, Web of Science, and Directory of Open Access Journals (DOAJ) and included relevant original articles, clinical trials, randomised controlled trials, case studies and case reports published in the last 7 years. In this review, we discuss the mechanistic rationale for CAR T-cell therapy in SLE, review the clinical outcomes reported to date, and explore its promise in ushering a new era of durable, drug-free remission in autoimmune disease.

## INTRODUCTION

Systemic lupus erythematosus (SLE) is a prototypical autoimmune disease characterised by heterogeneous clinical manifestations and multisystem involvement.^1^ Over the past several decades, there has been an overall improvement in SLE outcomes. The 5-year mortality rate, which was 50% in the 1950s, decreased to less than 5% by the 1990s, thanks to significant advances in immunosuppressive therapy and supportive care.^2,3^ A major breakthrough in lupus management came with the introduction of cyclophosphamide, originally developed for oncology.^4^ Transitioning from oral to intravenous administration preserved its therapeutic benefits while minimising toxicity.^5^ This was followed by the adoption of mycophenolate mofetil (MMF), a safer and equally effective oral immunosuppressant, particularly for lupus nephritis.^6^ The advent of biologic therapies in the 1990s, most notably rituximab (RTX), marked the beginning of targeted therapy in SLE.^7^ RTX, a B-cell depleting monoclonal antibody, has been used to rescue patients with refractory disease. These therapeutic advances, combined with a better understanding of lupus immunopathogenesis and improved general health-care, have contributed to reduced mortality.

However, despite this progress, the life expectancy of patients with SLE remains approximately 10 years shorter than that of the general population.^8^ This persistent gap is largely attributed to irreversible organ damage, heightened risk of infections, long-term cardiovascular complications, and increased malignancy risk.^9^ Current therapies are largely non-specific, where they suppress both pathogenic and protective immune cells, thereby increasing susceptibility to infections and cancer. Moreover, these treatments often fail to fully control the underlying immune dysregulation that drives cumulative organ damage in SLE. Even targeted therapies such as monoclonal antibodies frequently fall short in addressing the pleomorphic and redundant immunological networks involving T cells, B cells, NK cells, plasma cells, complement pathways, and neutro-phil extracellular traps.^10^

The emergence of chimeric antigen receptor (CAR) T-cell therapy, originally developed as a salvage strategy for relapsed hematologic malignancies, has generated enthusiasm for its application in autoimmune diseases.^11^ Building on the success of CAR T-cells in refractory blood cancers, researchers have begun exploring their utility in SLE, systemic sclerosis (SSc), idiopathic inflammatory myopathies (IIM), and other autoimmune conditions.^12^ A pivotal preclinical study by Kansal et al. (2019), demonstrated that CD19-targeted CAR T-cells induced sustained B-cell depletion and effectively reversed disease in a murine model of lupus, laying the foundation for clinical translation.^13^

Although CAR T-cell therapy has been extensively explored in oncology and discussed in general immunotherapy reviews, a focused review of its emerging role in systemic lupus erythematosus—integrating clinical outcomes, autoantibody biology, and target-specific mechanistic considerations—has been lacking in the rheumatology literature. In this narrative review, we aim to summarise the current evidence on CAR T-cell therapy in SLE, from preclinical studies to emerging clinical trials, highlighting its potential to redefine the therapeutic landscape of this complex autoimmune disease.

## METHODOLOGY

A comprehensive literature search was conducted in Medline/PubMed, Scopus, Web of Science, and Directory of Open Access Journals (DOAJ) from August 2018 to August 2025. The search strategy combined Medical Subject Headings (MeSH) and free-text keywords related to CAR T-cell therapy and systemic lupus erythematosus. The following Boolean search string was used. “Chimeric Antigen Receptor T-Cell Therapy”[MeSH] OR “CAR-T” OR “CAR T” OR “CAR T-cell” OR “CAR T cell therapy” OR “chimeric antigen receptor” AND “Lupus Erythematosus, Systemic”[MeSH] OR “systemic lupus erythematosus” OR “SLE” OR “lupus”.

We included relevant original articles, clinical trials, randomised controlled trials, case studies and case reports published in the last 7 years. The focus was on studies related to the treatment, outcome, and prognosis of CAR T-cell therapy in SLE.

We excluded:

- Animal studies
- Studies with incomplete data
- Duplicate reports
- Unpublished data (including congress abstracts)
- Review articles
- Non–peer-reviewed articles
- Commentaries and expert opinions
- Editorials

**Figure 1.**
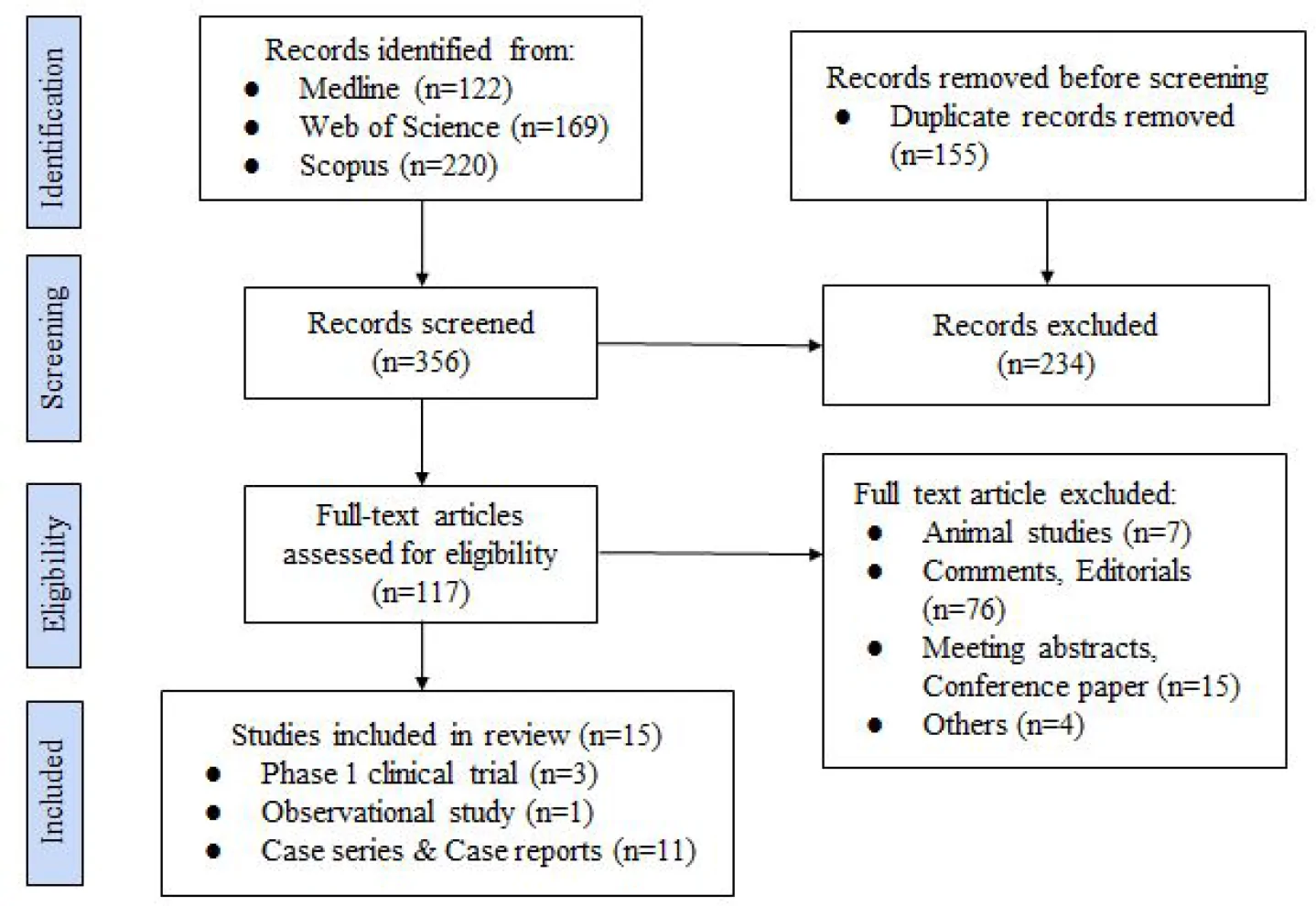
Flow diagram showing the selection process of studies included in the narrative review on CAR T-cell therapy in SLE.

After exclusion, 15 studies were included in the final review as summarised in **Figure 1**.

## WHAT ARE CAR T-CELLS?

CAR T-cells are specially engineered T-cells designed to attack specific targets. They are most often CD4+ or CD8+ T-cells that have been modified to express a chimeric antigen receptor (CAR) on their surface.^14^ The “chimeric” part means that the receptor is partly natural (from the original T-cell receptor) and partly synthetic (engineered to recognise a specific antigen). This receptor is introduced into the T-cell using a lentiviral vector.

In practice, the process works as follows:

- T-cells are collected from the patient through a procedure called leukapheresis.
- These T-cells are sent to a laboratory.
- In the lab, they are activated, purified, and transduced with the CAR gene using a lentivirus.
- The modified T-cells are expanded in number.

The cells are cryopreserved and sent back for infusion. Before infusion, the patient receives lymphodepleting chemotherapy (usually fludarabine and cyclophosphamide).

**Figure 2.**
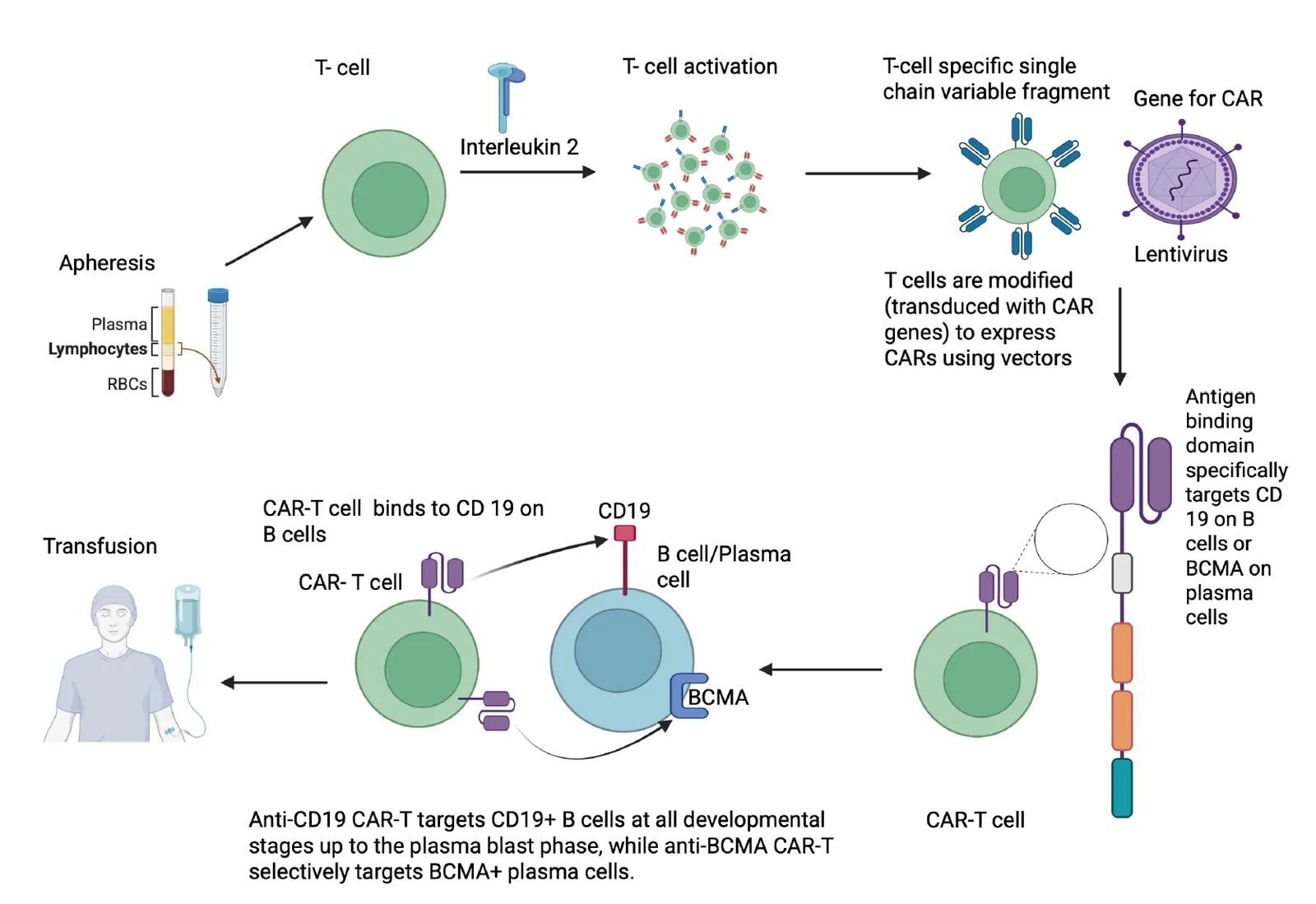
Schematic overview of CAR T-cell production.

The CAR T-cells are then thawed and infused intravenously (see **Figure 2**).

CAR T-cells have evolved over five generations. First-generation CARs included only a CD3ζ signalling domain, resulting in limited efficacy.^15,16^ Second-generation CARs added a single co-stimulatory domain (like CD28 or 4-1BB) for better activation and persistence, while third-generation CARs included two co-stimulatory domains for even greater function.^17–19^ Fourth-generation CARs (TRUCKs) are engineered to secrete cytokines, enhancing immune recruitment, and fifth-generation CARs incorporate cytokine receptor elements for full T cell activation via the JAK-STAT pathway.^20,21^ Each generation has aimed to boost potency, durability, and safety.

### Possible CAR T-Cell Targets in SLE

In systemic lupus erythematosus (SLE), B-cell lineage cells, including plasma cells, play a central role in autoantibody production and immune dysregulation. Among the cellular targets explored for CAR T-cell therapy in SLE, CD19 and BCMA (B-cell maturation antigen) are the most clinically relevant.

### CD19-Directed CAR T Cells

**Figure 3.**
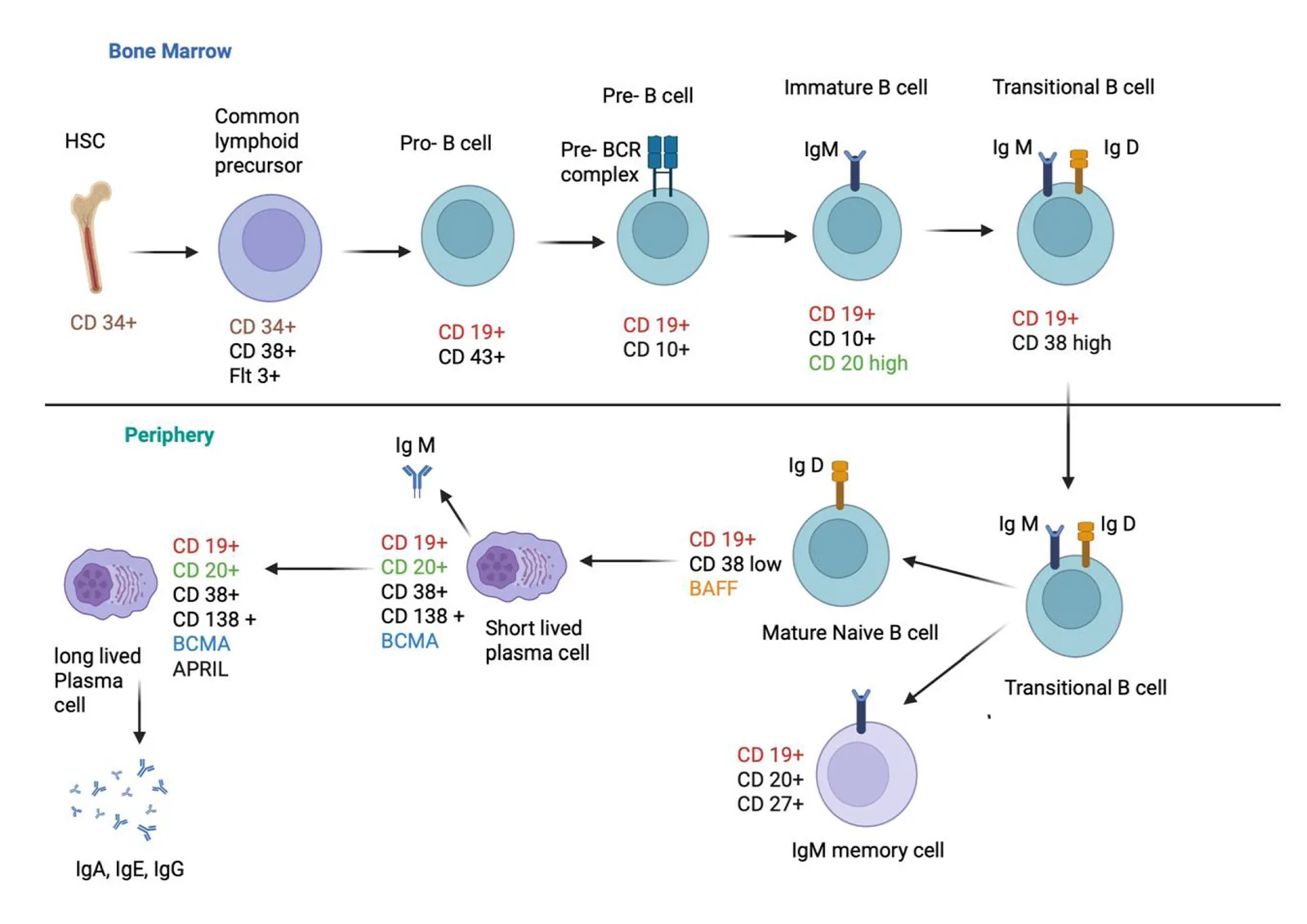
B cell development and B cell surface markers focusing CD19, CD20 and BCMA expression.

CD19 is expressed early and persistently across most stages of B-cell development, from pro-B cells to memory B cells (**Figure 3**). Its expression diminishes on plasmablasts and is absent on both short-lived and long-lived plasma cells. Consequently, CD19 CAR T-cell therapy targets a broad range of CD19-expressing B-cell populations, overlapping with but not identical to the B-cell compartment affected by rituximab. Importantly, CD19 CAR T-cells induce a deeper and more sustained B-cell depletion.^22^ However, long-lived plasma cells—lacking CD19 expression—remain unaffected and may continue to produce pathogenic autoantibodies. Therefore, CD19 CAR T-cell therapy induces consistent reduction in anti-dsDNA antibody titres, reflecting depletion of short lived plasmablasts and recently activated CD19 positive B cells. In contrast, autoantibodies against ribonucleoproteins which are predominantly produced by long lived CD19 negative plasma cells may persists despite CD19 directed CAR T-cell therapy.

### BCMA-Directed CAR T Cells

BCMA is expressed exclusively on antibody-secreting cells, including plasmablasts (expression begins here), short-lived and long-lived plasma cells (moderate to high expression). Targeting BCMA directly depletes the primary autoantibody source, a feature not addressed by either CD19 CAR T-cells or rituximab. This makes BCMA, a promising therapeutic target for autoantibody-driven forms of SLE, particularly refractory lupus nephritis or neuropsychiatric lupus where plasma cell activity is central.^23,24,25^

RTX, the commonly used rescue therapy in SLE, does not act against plasma cells. Moreover, RTX requires host mechanisms [e.g., Antibody dependent cellular cytotoxicity (ADCC), Complement dependent cytotoxicity (CDC), Direct CD20 mediated apoptosis] for depletion, which can be impaired in SLE.^26^ Additionally, study by Tur et al, showed that treatment with glycoengineered CD20 monoclonal antibody obinutuzumab and CD19/CD3 T cell engager blinatumomab do not consistently deplete B cells in the lymph nodes as compared to CAR T-cells.^27^ The targets of CAR T-cells in comparison with RTX are shown in **Table 1**.

**Table 1.** Comparison of Rituximab versus CAR T-cell therapies.

**Characteristic features**	**Rituximab (anti-CD20)**	**CD19 CAR T-cell therapy**	**BCMA CAR T-cell therapy**
Plasma cells	No	Absent on plasma cells, minimal effect on plasmablasts,	All plasma cells, Plasmablasts
Memory B cells	Yes	Yes	No
Early B cells	No	Yes	No
Autoantibody reduction	Partial	Moderate	Profound
B cell reconstitution	Yes (usually 4–6 months)	Sustained (beyond 6 months)	Sustained (beyond 6 months)
Mechanism of action	Antibody dependent cellular cytotoxicity (ADCC) Complement dependent cytotoxicity (CDC), Direct CD20 mediated apoptosis^26^	Perforin–granzyme– dependent apoptosis Fas/FasL–mediated cell death Cytokine-induced cytotoxicity	Perforin–granzyme– dependent apoptosis Fas/FasL–mediated cell death Cytokine-induced cytotoxicity

### CAR T-cell in SLE

Most of the data so far are in the form of case series and case reports as shown in table 2. Almost all have consistently shown that CAR T-cell therapy can induce rapid, deep, and sustained clinical and serological remission in patients with severe, refractory SLE. Drug-free remission after a single CAR T-cell infusion has been demonstrated for a maximum follow-up of 2 years.

**Table 2.** Studies on CAR T-cell therapy in SLE.

**Source (Year) /Authors**	**Patient Population /Case Details**	**Previous Immunosuppression**	**Pre-CAR T Disease Activity (SLEDAI/BILAG)**	**Post-CAR-T Disease Activity**	**Duration of Follow-up**	**Infections & Other Side Effects**
**Mougiakakos et al. (2021)^22^**	Refractory SLE, 1 Patient	Median 5 agents	SLEDAI-16	SLEDAI-0	3 months	No severe infection; mild fever, no CRS/ICANS
**Mackensen et al. (2022)^28^**	5 refractory SLE	Median 5 agents	SLEDAI median 16	SLEDAI 0	Median-13 months (4–22 months)	No severe infections; mild CRS (grade 1–2), transient cytopenia
**Feng et al (2023)^29^**	12 refractory SLE, CD19/BCMA CAR-T	Multiple, refractory	SLEDAI-2K Mean 18.3	SLEDAI-2K Mean 1.5	3–6 months	No severe infection; all had grade 1 CRS, manageable cytopenia
**Müller et al. (2024)^30^**	15 autoimmune (8 SLE, 3 myositis, 4 systemic sclerosis) ^*^	Median 5 agents	SLEDAI median 13 (Range 9.3–16)	SLEDAI 0	Median 17 months (range 6–29 months)	No severe infections; mild CRS, transient cytopenia
**Li et al. (2024)^31^**	1 SLE patient with refractory ITP	5 agents	Severe thrombocytopenia (<20×10^9^/L )^#^	Platelet 109,000 by 6 months	6 months	No severe infections; no CRS/ICANS
**Hagen et al. (2024)^32^**	Single case, CNS SLE	Multiple, refractory	CNS symptoms, SLEDAI-22	Resolution of CNS SLE, SLEDAI -0	3 months	No severe infections; mild CRS
**Wang et al. (2025)^33^**	3 refractory SLE, allogeneic CAR T-cell therapy	Multiple, refractory	SLEDAI Median 10 (range 10–14)	SLEDAI median 0 (Range 0–2)	12 months	No severe infections; mild CRS, transient cytopenia
**Shu et al (2025)^34^**	Open label, phase 1, dose escalation study /8 moderate to severe SLE	Multiple, refractory	SLEDAI Mean 11.75	SLEDAI Mean 1.625	6 months	mild CRS; no ICANS; only one patient had severe infections & complications like HLH, managed and recovered
**Ziwei et al (2025)^25^**	open-label, single-arm clinical trial in refractor LN, using anti-BCMA CAR T cells	Multiple, refractory	SLEDAI median 18 (range 10–22	SLEDAI 0 (range 0–4 )	9 months	no ICANS or severe infections, only one case of grade I CRS

### Possible side effects of CAR T-cells

The most notable side effects associated with CAR T-cell therapy include cytopenias, infections, cytokine release syndrome (CRS), and immune effector cell-associated neurotoxicity syndrome (ICANS). CRS occurs when activated CAR T cells encounter their target antigen, leading to rapid proliferation and a massive release of inflammatory cytokines such as IL-6, IFN-γ, and TNF-α. This cytokine surge triggers a systemic inflammatory response, which can range from mild fever to life-threatening multi-organ dysfunction.^35^ ICANS, on the other hand, is believed to result from cytokine-mediated disruption of the blood-brain barrier and direct neuro-inflammatory effects, manifesting as confusion, seizures, or encephalopathy.^36^ Both CRS and ICANS are closely linked to the intensity of immune activation and the degree of cytokine release following CAR T-cell infusion.

Interestingly, the incidence and severity of these adverse events appear to be significantly lower in SLE patients compared to those undergoing CAR T-cell therapy for malignancies.^37^ This is likely due to the lower doses of CAR T-cells used in autoimmune disease trials, where the goal is immune modulation rather than eradication of malignant clones. Similarly, haematological side effects including cytopenias are less frequently observed with CAR T-cell therapy in SLE patients.^38^ In the landmark study by Müller et al., only one patient (Patient 8) experienced cytopenia, hypogammaglobulinemia, and a respiratory infection that required inpatient care.^30^ However, based on personal email communication with the authors, all patients remain in remission with no major adverse events reported during on-going follow-up.

Recently, Hagen et al. reported a new side effect in autoimmune diseases following CAR T-cell therapy and coined it LICATS-local immune effector cell-associated toxicity syndrome. LICATS is usually a mild, self-limited, organ-specific side effect frequently observed in autoimmune patients. This is different from CRS and does not resemble an autoimmune flare. LICATS is a time-limited event associated with B-cell depletion, and regress spontaneously or after a short course of treatment with glucocorticoids.^39^

## UPCOMING TRIALS

Following the initial reports of sustained clinical and serological remission, there is a global surge in CAR T-cell research for SLE, with a focus on different CAR constructs (CD19, BCMA, dual-target), sources (autologous, cord blood), and safety/efficacy as primary endpoints. Most are in the early phase, with completion expected between 2025–2028. To the best of our knowledge, no large phase 3 trials have yet been registered. Summary of the on-going and upcoming CAR T-cell trials in SLE, from major medical databases (e.g., ClinicalTrials.gov, WHO ICTRP) is summarised in Table 3.^40^

**Table 3.** Selected on-going clinical trials of CAR T-cell therapy trials in SLE, retrieved from ClinicalTrials.gov (accessed August 31, 2025).

**NCT Number**	**Study Status**	**Intervention**	**Phase**	**Start Date**	**Completion Date**	**Location(s)**
NCT06297408	Not yet recruiting	Relma-cel (CD19 CAR T-cell)	Phase 1	Mar 2024	May 2026	Shanghai Ming Ju Biotechnology Co., Ltd (Peking Union Medical College Hospital)
NCT05765006	8 patients Recruited	Relma-cel (CD19 CAR T-cell)	Phase 1	Feb 2023	Not specified	Multicentre study, sponsored by JW Therapeutics, Shanghai
NCT06681337	Not yet recruiting	BCMA and CD19 CAR-T-cell	Phase 1	Nov 2024	Dec 2025	Bioray Laboratories, affiliated to Nanjing University Medical School.
NCT06038474	Recruiting	Descartes-08 BCMA targeted (Autologous mRNA CAR T-cell therapy	Phase 2	Feb 2024	Nov 2026	Therapeutics, Cartesian California
NCT03030976	Unknown	CD19 CAR T-cell	Phase 1	Mar 2017	Not specified	Shanghai GeneChem Co., Ltd
NCT06462144	Recruiting	IMPT-514 (autologous CD19/CD20 CAR T-cell )	Phase 1	Jun 2024	Oct 2026	Immunophage Bomedical, affiliated to Nanjing University Medical School, Shanghai
NCT06549296	Recruiting	RD06-04 (CD19 CAR T-cell)	Phase 1	Sep 2024	Aug 2027	Nanjing Bioheng Biotech Co., Ltd, Xuzhou Medical University affiliated hospital, Jiangsu
NCT06310811	Recruiting	RD06-04, at two escalating dose levels (CD19 CAR T-cell)	Phase 1	Mar 2024	Mar 2027	Union Hospital, Tongji Medical College, Wuhan
NCT06947460	Recruiting	CD19-BCMA CAR T-cell	Phase 1/2	Apr 2025	Jul 2026	Beijing GoBroad Hospital, China
NCT06947473	Not yet recruiting	Umbilical Cord Blood CD19-BCMA CAR T-cell	Phase 1/2	May 2025	Jul 2026	Beijing GoBroad Hospital, China
NCT06503224	Recruiting	BCMA CAR T-cell (autologous)	Phase 1	Apr 2024	Apr 2028	Hunan Siweikang Therapeutic Co., Ltd, Changsha, Hunan, China

## LIMITATIONS AND CONCERNS

Despite encouraging early results, several limitations need to be considered when evaluating CAR T-cell therapy for SLE. Although B cells are central to lupus pathogenesis, other immune cells such as T cells, NK cells, and neutrophils also contribute. Solely targeting B cells may not be sufficient for all patients, particularly those with disease driven by non B-cell pathways. Most published studies have follow-up periods of only 6 months to 2 years. While remission rates are promising, the long-term durability of response, potential for late relapses, and delayed toxicities remain unknown. Many patients with severe or treatment-resistant SLE may have lymphopenia due to prior immunosuppression. It may be a challenge to collect enough functional autologous T cells for CAR T-cell manufacturing. Current CAR T-cell protocols require discontinuation of all immunosuppressive drugs, including steroids, before CAR T-cell infusion. This drug-free window may not be feasible in patients with active, life-threatening disease, as it could trigger flares. While infection rates in early trials have been low, most studies come from low tuberculosis prevalence settings. In tuberculosis-endemic countries, profound B-cell depletion may increase the risk of reactivation or opportunistic infections. In addition, cost, affordability and availability may be a concern, especially in resource-limited settings.

## CONCLUSION

By consolidating early clinical evidence and mechanistic insights specific to SLE, this review aims to provide rheumatologists with a conceptual framework for understanding how CAR T-cell therapy may fit into future treatment strategies for refractory disease. Overall, CAR T-cell therapy in SLE remains in its nascent stages, with current evidence limited to small patient cohorts and the absence of randomised controlled trials. More robust studies are needed to determine its long-term safety, efficacy, and comparative effectiveness against existing standard-of-care therapies. Nevertheless, the prospect of achieving sustained clinical and serological remission—or even a potential cure—with a single infusion of CAR T-cells offers a compelling paradigm shift. The ability of infused CAR T-cells to retain immunological memory raises the possibility of preventing de novo autoantibody generation and promoting durable, drug-free remission. This emerging strategy offers a significant source of hope for both rheumatologists and patients living with lupus.
